# Double Pituitary Adenomas in Patients With Cushing’s Disease

**DOI:** 10.7759/cureus.38923

**Published:** 2023-05-12

**Authors:** Oleksandr Voznyak, Nazarii Hryniv, Andrii Lytvynenko, Yaroslav Zinkevych

**Affiliations:** 1 Centre of Neurosurgery, Clinical Hospital "Feofaniya", Kyiv, UKR

**Keywords:** endocrinology, multiple adenomas, transsphenoidal microsurgery, cushing’s disease, pituitary adenoma

## Abstract

Background and objective

Double and multiple pituitary adenomas (PAs) are discovered in 2.6-3.3% of patients with Cushing’s disease and in approximately 1% of autopsies. Undiagnosed and unremoved second PA may be the cause of unsuccessful surgical treatment of Cushing’s disease. In this study, we aimed to describe our experience with the detection and treatment of patients with double PAs.

Methods

All patients in our series underwent transsphenoidal surgery (TSS) with endoscopic and neuronavigation assistance. Before 2017, we completely relied on MRI findings while planning surgery. From 2017 onwards, a broad revision of the sella turcica was conducted during surgeries regardless of the MRI data.

Results

Overall, 81 patients were included in the study: 51 before 2017 and 30 in 2017 after. In the pre-2017 group of patients, three out of 51 had double adenomas, and all of them were seen on MRI images. We encountered four more double PAs during the next period. Only two of them had been predicted by MRI. The remission rate was higher after 2017 and amounted to 90% (27 out of 30 patients). In contrast, before the implementation of total revision (pre-2017), our success rate was 82% (42 out of 51 cases). Both neoplasms in cases of double PAs yielded similar histological and immunohistochemical (IHC) features but were consistent with multiple PAs.

Conclusions

Although the improvement in our results in recent years cannot be clearly attributed to a targeted search for the second microadenoma, we still recommend performing a broad inspection of the sella turcica after the excision of the pituitary microadenoma regardless of preoperative MRI data.

## Introduction

Human adenohypophysis consists of three parts: pars distalis, pars intermedia, and pars tuberalis. Hormone-secreting cells are discreetly located in separate groups in the pars distalis [1]. Most researchers accept the monoclonal theory of pituitary adenoma (PA) tumorigenesis [2,3]. However, simultaneous growth of separated pituitary tumors with different secretory activity is possible [4]. Double and multiple PAs have been discovered in 2.6-3.3% of patients with Cushing’s disease and in approximately 1% of autopsies [5,6,7]. The ambiguity around the use of the terms “double” vs. “multiple” stems from a lack of consistency in the literature. Some authors require tumors to be topographically unique to qualify them as separate, and others insist on separate immunohistochemistry (IHC) and/or genetic definition [8,9]. If two PAs are detected, it is impossible to estimate the secretory activity of each of them using MRI [10]. The reported diagnostic efficacy of inferior petrosal sinus sampling (IPSS) is 82-100% across studies [11]. However, this method has its own limitations and peculiarities in practice. There is an obvious need to obtain more clinical data about these lesions, as treatment decisions rely on accurate clinical assessments. In light of this, we conducted a retrospective case-control study on the investigations and treatment of double adrenocorticotropic hormone (ACTH)-secreting PA in patients with Cushing’s disease. The purpose of the study was to describe our experience with the detection and treatment of patients with double PAs.

## Materials and methods

This retrospective case-control study was conducted at our institution by evaluating patients’ records from 2010 to 2019. All surgeries were performed by one senior neurosurgeon. The inclusion criteria were as follows: male and female patients older than 18 years with histologically verified ACTH-secreting PA. All the included patients had undergone surgery for the first time. All double PA cases were confirmed by obtaining the immunohistological result of two separate samples from non-connected pituitary lesions. We excluded patients with incomplete tumor removal based on postoperative MRIs. If another source of ACTH except for adenoma was found during pre- or postoperative investigations, the case was excluded from the study. All patients received complete clinical, neurological, and ophthalmological examinations and underwent transsphenoidal microsurgery (TSS) with endoscopic and neuronavigation assistance.

We used T1-weighted, 0.8-1-mm, dynamically contrast-enhanced MRI (1.5 Tesla) to assess tumors and adjacent structures. All procedures were performed by the same team of neurosurgeons. Gross total resection (GTR) was defined as a complete tumor removal confirmed by the surgeon intraoperatively and no evidence of residual tumor on the postoperative MRI at three and 12 months postoperatively. Biochemical remission of Cushing’s disease was defined as the restoration of healthy plasma cortisol levels two weeks after the surgery (i.e., plasma cortisol values <54 ng/mL at midnight and 50% of the values at 8:00 AM), and normal 24-hour urinary cortisol levels.

All patients had at least 12 months of follow-up in coordination with the endocrinologist. Those who did not achieve remission were referred to an endocrinologist for further investigations. IHC examination of tumor specimens was performed in all cases. 

The null hypothesis was as follows: revision of both halves of the adenohypophysis during the surgical procedures regardless of MRI data is not associated with better outcomes when compared with standard excision of only MRI-visible PA in patients with Cushing’s disease. The alternative hypothesis was as follows: treatment of patients with ACTH-secreting PA will lead to improved outcomes if both halves of the adenohypophysis are revised during the surgical procedures regardless of MRI data.

Statistical analyses were conducted using IBM SPSS Statistics for Windows, Version 23.0. (IBM Corp., Armonk, NY). The quantitative data were tested for normality. The mean of numeric variables between the two groups was compared with independent-samples two-tailed t-tests. The distributions of categorical variables between the two groups were compared using the Chi-squared test.

Surgical technique

We performed a standard microscopic transsphenoidal approach. The sella turcica was perforated widely horizontally between the cavernous sinuses and vertically from the planum sphenoidale to the clivus. We used the H-shaped incision in the dura mater, which enabled good visualization of the entire anterior surface of the pituitary gland. After the PA was detected (always lateralized), it was removed using microsurgical dissectors, cup curettes, ring curettes, and an aspirator. Broader adenohypophysis revision began with the examination of its surface, followed by curettage of the space between the medial wall of the cavernous sinus and the surface of the pituitary gland. If a second PA was detected, it was removed in the same way as the first, and both adenomas were sent for histopathological diagnosis separately.

Histopathological diagnosis of pituitary adenoma

For PA classification, the specimens were cut and stained with hematoxylin and eosin (H&E); IHC staining with monoclonal antibodies against the specific pituitary hormones, including growth hormone (GH), prolactin (PRL), and ACTH, was carried out on the tumor tissues. In addition to Kі-67, somatostatin receptor 2 (SSTR2) expression was evaluated. Histopathological diagnosis was verified by experienced pathologists in line with the 2017 World Health Organization (WHO) classification [12].

## Results

**Figure 1:**
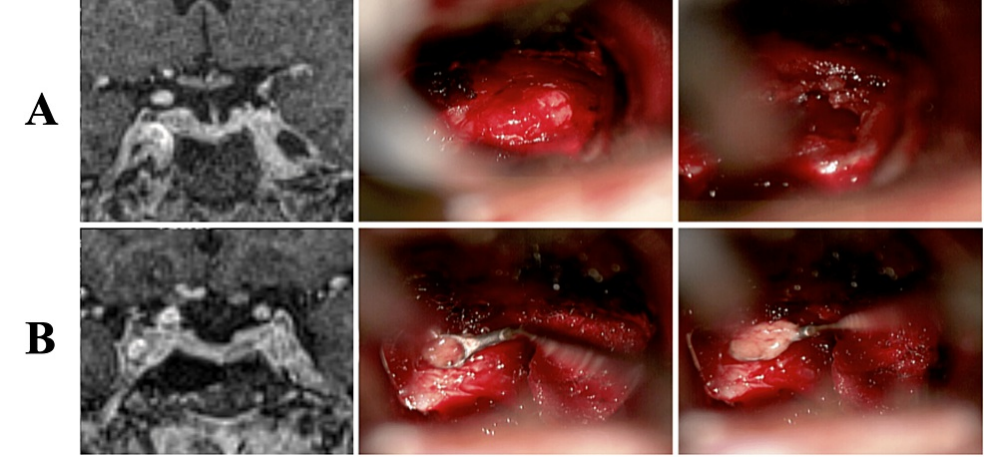
MRI from the navigation system and intraoperative images of patients with double pituitary adenomas (A) Right side. (B) Left side MRI: magnetic resonance imaging

Before 2017, we relied on MRI findings for surgery planning. From 2017 onwards, we started revising both halves of the adenohypophysis during surgical procedures regardless of MRI data. A total of 81 patients were included in the study: 51 before 2017 and 30 in 2017 and after. The clinical characteristics of patients are shown in Table 1. In the pre-2017 group of patients, three out of 51 had double adenomas, which were all seen on MRI images. In 2017 and beyond, we found four double PAs; only two of them had been predicted by MRI (Figure 1). Additional tumors were not seen in radiographs. Instead, we found them via wide inspection of the sella turcica in each case.

**Table 1:** Demographic, assessments, and outcomes data ACTH: adrenocorticotropic hormone; GTR: gross total resection; PA: pituitary adenoma

Variables	Before 2017	2017 and after	P-value
Number of patients	51	30	N/A
Age, years, mean ± SD	37 ± 13	42 ± 16	0.465
Female-to-male ratio	32/19	22/8	0.628
Preoperative ACTH plasma levels, ng/ml, mean ± SD	132 ± 76	133 ± 54	0.468
Preoperative cortisol plasma levels, ng/ml, mean ± SD	287 ± 191	307 ± 143	0.552
Tumor size, mm, median (IQR)	5.6 (2.5–8.3)	6.2 (2.3–9.7)	0.107
Patients with double PA, n (%)	3 (5.9%)	4 (13.3%)	0.294
Female-to-male ratio	2/1	3/1	0.871
Intraoperative finding of a second tumor, n (%)		2 (6.6%)	N/A
GTR	51	30	N/A
Remission, n (%)	42 (82%)	27 (90%)	0.349
Mean follow-up time, months	12	13	0.318

The remission rate was higher in 2017 and beyond (27 of 30 patients, 90%). For the pre-2017 MRI-only patients, the remission rate was 82% (42 of 51 cases). All the tumors in both groups were microadenomas. Of the nine patients treated before 2017 who did not achieve remission, three had GTR. None of the included patients had postoperative complications such as hemorrhage, diabetes insipidus, or CSF leakage.

**Figure 2:**
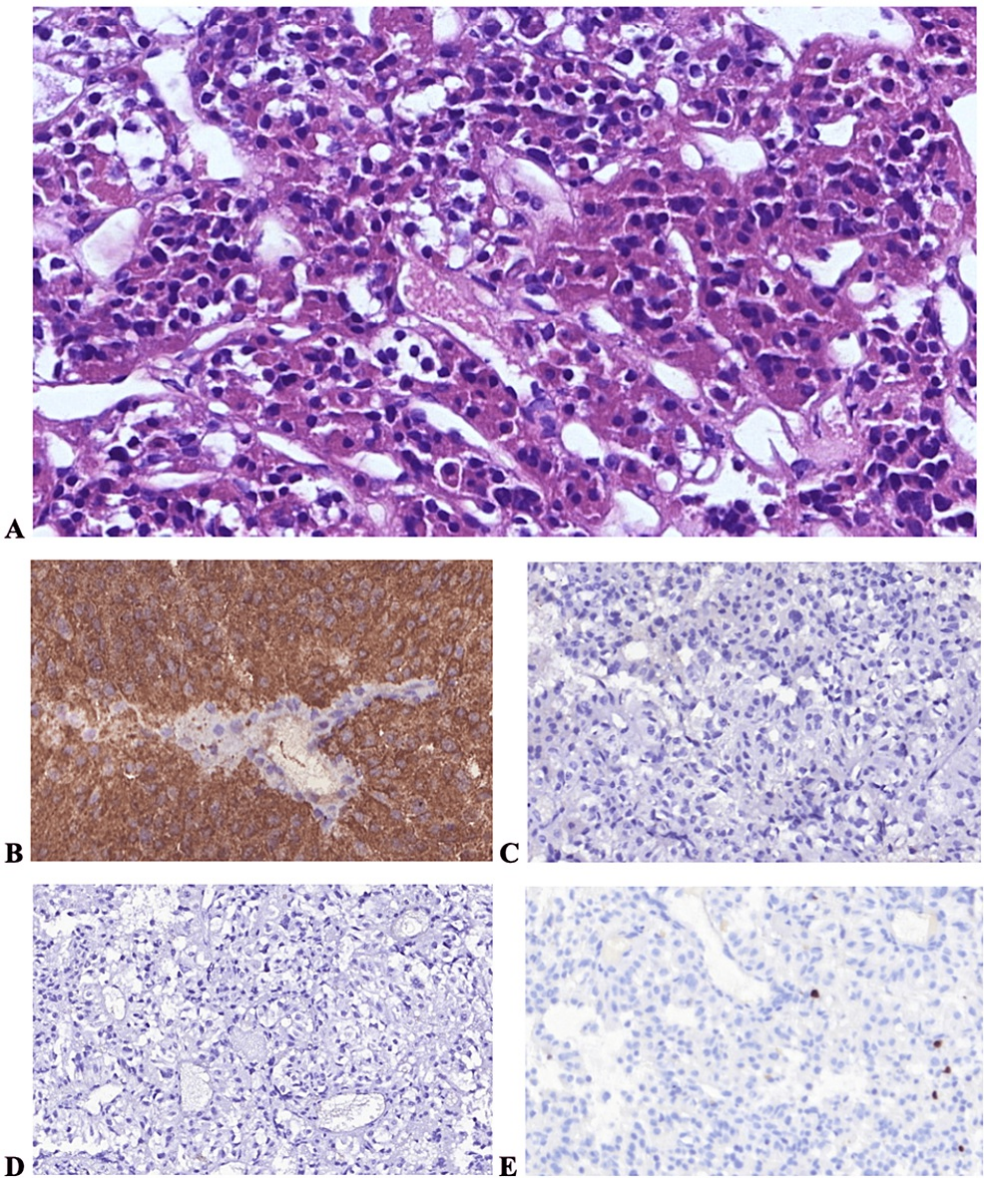
Histological features of the first pituitary adenoma The neoplasm demonstrated trabecular-like structures separated by large sinusoidal vessels. Trabeculae comprised numerous basophilic cells with some large acidophilic cells (A). Tumor cells demonstrated strong immunoreactivity for ACTH (B) and were negative for GH (C) and PRL (D). There were a few Ki-67-positive cells (E) ACTH: adrenocorticotropic hormone; GH: growth hormone; PRL: prolactin

**Figure 3:**
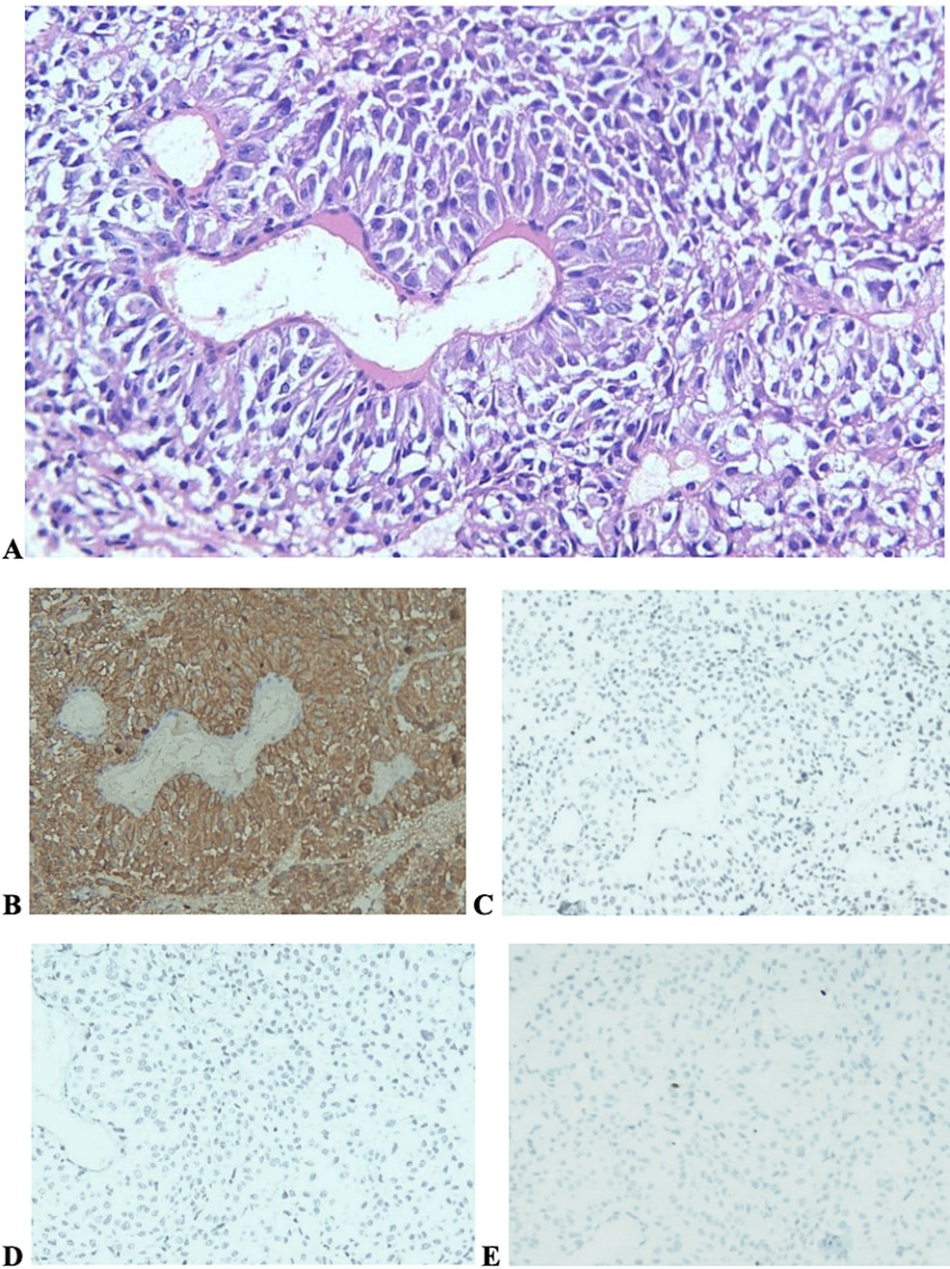
Histological features of the second pituitary adenoma The tumor demonstrated structural and IHC features similar to the first neoplasm. (A) Hematoxylin and eosin staining. (B-E) IHC staining for ACTH, GH, PRL, and Ki-67, respectively. Magnification ×200 ACTH: adrenocorticotropic hormone; GH: growth hormone; IHC: immunohistochemical; PRL: prolactin

Both neoplasms in all cases of double PAs usually yielded similar histological features (Figures 2, 3). Nevertheless, in all cases, the histopathological and IHC features were consistent with multiple PAs. The assessment of the prognostic marker Ki-67 revealed a positive reaction in 1-3% of tumor cells, reflecting a low probability for recurrence; however, the differences in this indicator could not be considered significant.

## Discussion

Both external influences on adenocytes and specific genetic determinations for mutations may lead to PA formation. Discrete collocation of differently active cells provides the possibility for the simultaneous occurrence of two or more PAs with a different secretory activity. In the case of double PAs, it will not be known for certain which is the biochemically important tumor until they are removed.

Along with the undiagnosed ectopic ACTH secretion, removing the “wrong” PA and leaving an active tumor could be among the reasons for the unsuccessful surgical outcomes in Cushing’s disease [13]. The isolated secretory activity of each of the two detected PAs in patients with Cushing’s disease could not be non-invasively determined before surgery. IPSS was found to have high accuracy; however, this surgical method is accompanied by additional risks and incomprehensibly large differences in terms of reported efficiency (54-100%) [14]. The adverse events reported include hematoma development in up to 4%, vasovagal reactions, subarachnoid hemorrhage (SAH), and medullary infarcts. Since double PA cases are rare, a lack of centers experienced in managing them contributes to higher risks related to IPSS [15]. In Ukraine, this procedure represents a marked additional financial load. These factors forced us to reject this method in regular practice.

Size, as well as growth peculiarities, are not predictive factors for the secretory activity of PA [16]. Even 2-mm-diameter tumors could cause Cushing’s disease with a concomitant inactive macroadenoma [17]. Some authors have indicated that the IHC examination findings for the secretory granules do not necessarily correlate with secretory activity [18,19,1]. An ectopic source of ACTH secretion could explain the failure to achieve remission after the excision of an MRI-visible tumor [20,21]. Before 2017, three of our patients had GTR but not biochemical remission. One had ectopic secretion, but the other two did not, possibly due to the undetected concomitant PA. According to the literature, physicians have an approximately 20% chance to detect undiagnosed PA during a surgical operation [22,12,13]. In addition, two PAs were investigated in our department by the intraoperative inspection of the sella turcica after 2017, comprising 50% (two of four) of all double PA cases in our study. Therefore, the broad inspection of the sella turcica cavity during surgery was justified and appropriate.

ACTH-secreting PAs are mostly lateralized, and while the broad adenohypophysis revision could assist in other PA findings it does not improve a thorough removal of a single PA. The proposed surgical maneuver could possibly be useful for the total removal of GH-secreting PAs, as they more often grow centrally and typically spread along the sella turcica walls.

The rarity of double PA cases prevents us from making definitive statistical conclusions. The varying definitions of “double adenomas” also had an influence on our examination of data elicited from the literature search.

## Conclusions

Although the improvement in our results in recent years cannot be clearly attributed to a targeted search for the second microadenoma, we still consider it appropriate to perform a broad inspection of the sella turcica after the excision of the pituitary microadenoma regardless of the preoperative MRI data. Our study was limited by the relatively small sample size, and the lack of sufficient data prevents us from making broadly applicable recommendations. We believe this article will raise awareness of and generate discussions around unsuccessful ACTH-secreting adenoma surgeries and the need to explore whether two separate tumors exist.
